# Pan-filovirus activity of an IGF2-fused monoclonal antibody: Impairment by IGF1R cross-engagement and rescue via IGF2 Y27L mutation

**DOI:** 10.1016/j.virusres.2026.199755

**Published:** 2026-05-25

**Authors:** Wen Hu, Xiaonan Wu, Yuting Zhang, Siyu Zhao, Junjie He, Xuanyi Li, Muyang He, Jing Wang, Longlong Luo, He Xiao, Xinying Li, Chenghua Liu, Weijin Huang, Youchun Wang, Chunxia Qiao, Jiannan Feng, Yan Wen, Guojiang Chen

**Affiliations:** a College of Biotechnology, Campus of Jiangsu University of Science and Technology, Zhenjiang 212100, China; b State Key Laboratory of National Security Specially Needed Medicines, Beijing, China; c Joint National Laboratory for Antibody Drug Engineering, Henan University, Kaifeng, China; d College of Life Sciences and Biopharmaceuticals, Shenyang Pharmaceutical University, Shenyang, China; e Wuya College of Innovation, Shenyang Pharmaceutical University, Shenyang 110016, China; f Division of HIV/AIDS and Sex-transmitted Virus Vaccines, National Institutes for Food and Drug Control, Beijing, China

**Keywords:** Filoviruses, Neutralizing antibody, IGF1R, IGF2R

## Abstract

**Background:**

Filoviruses (Ebola and Marburg) cause severe high-mortality infectious diseases. Neutralizing antibodies represent promising antiviral candidates, but broadly reactive antibodies against multiple filoviruses are rare. Antibody-ligand fusion strategies that combine conserved glycoprotein-targeting antibodies with IGF2R ligands can mediate endosomal antibody delivery. Previously, we showed that an NPC2-fused AF-03 (an antibody to the GP1 core region) exhibited pan-filovirus neutralization. However, this NPC2 fusion faced quality control challenges and its application is limited by heterogeneous glycosylation, batch inconsistency, and poor CMC feasibility.

**Methods:**

Human IGF2 or its Y27L variant was fused to the N-terminus of the AF-03 light chain via a peptide linker. Cellular attachment was measured by flow cytometry and ELISA. Internalization was assessed by flow cytometry, western blotting and confocal imaging. Neutralizing activity against pseudotyped filoviruses was determined using a luciferase reporter system in vitro.

**Results:**

The IGF2-fused antibody (termed AF03-IL) exhibited broad-spectrum neutralizing activity against multiple pseudotyped filovirus in vitro. However, its potency, including internalization and neutralization, was much weaker than that of the NPC2-fused antibody (termed AF03-NL). The reduced activity was caused by IGF2 binding to IGF1R on the cell surface. To overcome this limitation, we introduced a single point mutation (Y27L) into the IGF2 domain. This new construct, named AF03-IL_m1_, showed superior internalization and neutralization activity compared to the parental AF03-IL.

**Conclusions:**

The anti-filovirus activity of IGF2-fused AF-03 is impaired by cross-engagement with IGF1R that is rescued by Y27L introduction. The work also might have implications into the design of IGF2-based protein degradation molecules.

## Highlights

• Y27L mutation in IGF2 moiety enhances AF03-IL’s pan-filovirus neutralization by unleashing cross-engagement with IGF1R.

## Graphical abstract

**Figure:**
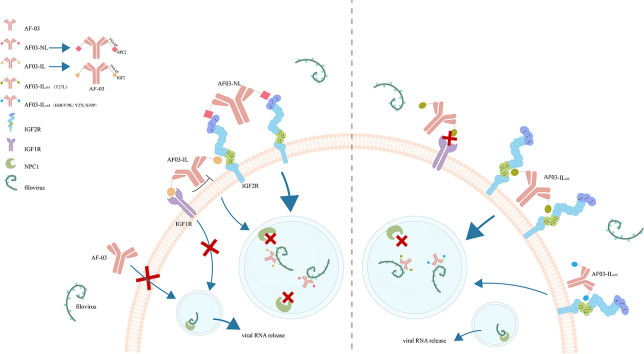

## 1 Introduction

Filoviruses are single-stranded, negative-sense RNA viruses. This class primarily includes *Ebolavirus* and *Marburgvirus*. The *Ebolavirus* genus comprises Ebola virus (EBOV), Sudan virus (SUDV), Bundibugyo virus (BDBV), and Tai Forest virus (TAFV). The *Marburgvirus* genus includes Marburg virus (MARV) and Ravn virus. These viruses are highly lethal in humans. Reston virus (RESTV) is an exception (Kuhn et al., 2013; Kuhn et al., 2010; Rougeron et al., 2015). In the past decade, several novel filoviruses have been discovered. These included Llocviu virus (LLOV), Bombali virus (BOMV), and Měnglà virus (MLAV). Their risk to humans remains unknown (Goldstein et al., 2018; Negredo et al., 2011; Maruyama et al., 2014; Ng et al., 2014; Yang et al., 2019). Many therapeutics have been tested in clinical trials (Liu et al., 2017; Mulangu et al., 2019). Among these, monoclonal antibody (mAb)-based therapies targeting the EBOV glycoprotein (GP) are considered the most promising. A three-antibody cocktail (REGN-EB3) and a single antibody (mAb114) have been approved for EBOV disease treatment (Pascal et al., 2018; Corti et al., 2016). Several pan-ebolavirus antibody combinations have shown cross-neutralization and protection against EBOV/SUDV/BDBV in pre-clinical studies, including MBP134^AF^ (Wec et al., 2019; Bornholdt et al., 2019), rEBOV-442/ rEBOV-515 (Gilchuk et al., 2021), 1C3/1C11 (Milligan et al., 2022), and CA45/FVM04 (Brannan et al., 2019). However, these mAbs lack broad antiviral activity. They cannot block infection by other filoviruses. Filovirus outbreaks are sporadic. It is difficult to predict which viral species will cause the next outbreak. Therefore, broadly protective anti-filovirus mAb therapeutics are highly desirable.

A hallmark of filoviruses cell entry is the proteolytic cleavage of GP (GP_CL_) in the late endosome/lysosome. This cleavage uncovers “cryptic” epitopes. Among these is the receptor-binding site (RBS). The RBS engages with an intracellular receptor called Niemann-Pick C1 (NPC1) (Carette et al., 2011; Miller et al., 2012; Côté et al., 2011). This engagement is indispensable for cell entry and infection by all filovirus species (Ng et al., 2014; Miller et al., 2012; Spence et al., 2016). Therefore, targeting the GP_CL_-RBS interface is a reasonable strategy for developing pan-filovirus immuotherapeutics. However, this interaction occurs intracellularly. It is not accessible to conventional antibodies. To address this obstacle, we and others recently developed a “Trojan horse” antibody strategy. This approach serves as a complementary method for generating cross-protective antibodies (Wec et al., 2016; Wirchnianski et al., 2021; Zhang et al., 2024). In a pioneering strategy, Chandran and colleagues engineered a bispecific antibody by coupling one monoclonal antibody targeting the NPC1 or RBS domain with another monoclonal antibody that recognizes a surface-exposed GP epitope. This design hijacks viral particles to achieve endosomal delivery, enabling broad neutralization to all known ebolaviruses and providing post-exposure protection against multiple ebolaviruses strains in mouse models (Wec et al., 2016). More recently, they developed a second-generation Trojan horse antibody. In this design, a ligand peptide is either NPC2 or insulin growth factor 2 (IGF2). Both ligands bind to the host cation-independent mannose-6-phosphate receptor (CI-MPR), also known as IGF2R. This cargo harnesses endocytosis triggered by the NPC2/IGF2-CI-MPR interaction. It transports itself into the endo/lysosome and then blocks viral and host membrane fusion with pan-filovirus breadth (Wirchnianski et al., 2021). We screened a novel antibody named AF-03 using phage display technology. This antibody targets the NPC1-binding domain of MARV GP and exhibits neutralizing and protective activities against pseudotyped MARV both in vitro and in vivo. We further fused NPC2 to the N terminus of the AF-03 light chain to generate AF03-NL. This fusion protein achieves neutralization against multiple filovirus species and EBOV mutants through binding to CI-MPR (Zhang et al., 2024). Notably, mannose-6-phosphate (Man6P) motifs attached to asparagine residues in NPC2 are indispensable for endocytosis upon NPC2-CI-MPR ligation (Chikh et al., 2004; Liou et al., 2006), However, this post-translational modification presents manufacturing challenges. The modification is heterogeneous. It causes batch-to-batch inconsistency. These issues create CMC limitations in drug development and production. Therefore, we propose IGF2 as a replacement for NPC2. IGF2 is a non-glycosylated ligand. It mediates endosomal delivery by binding to domain 11 of CI-MPR (Brown et al., 2008; Zaccheo et al., 2006). In this work, we generated an IGF2-fused AF-03 (termed AF03-IL). IGF2 was attached to the N terminus of the light chain. We then evaluated its cellular internalization and neutralization capacity. Unexpectedly, AF03-IL showed much weaker endo/lysosomal trafficking than AF03-NL upon incubation with host cells. Consistently, AF03-IL exhibited weak neutralizing activity against ten filovirus species and three EBOV mutants in a pseudovirus system. Further mechanistic exploration confirmed that cell surface IGF1R competed for IGF2 binding, interfered with the interaction between AF03-IL and CI-MPR, and thus impaired cellular internalization (LeRoith et al., 2021). To overcome this competitive binding limitation, we introduced a classic Y27L point mutation into the IGF2 domain of AF03-IL. First reported in 1991 via site-directed mutagenesis, the Y27L mutation has a well-established structural basis for receptor selectivity (Bürgisser et al., 1991). The surface-exposed IGF2 Tyr^27^ residue mediates high-affinity IGF1R binding through aromatic stacking. This mutation specifically abolishes IGF1R binding while preserving CI-MPR affinity, rewiring IGF2 receptor preference to eliminate IGF1R competitive inhibition and restore the endolysosomal trafficking and neutralization activity of the fusion protein.

## 2 Materials and methods

### 2.1 Protein preparation

Chinese Hamster Ovary Suspension (CHO-S) cells were purchased from American Type Culture Collection (ATCC) and cultured in ExpiCHO Expression Medium (Gibco, Cat. No. A2910001) in an orbital shaker incubator (Kuhner) at 37 °C, 8% CO_2_. The human NPC2 gene (GenBank: NP_001350617.1, aa20-151) and the human IGF2 gene (GenBank: NP_000603.1, aa25-91) was linked at N-terminus to the variable domain of the light chains of AF-03 via a short amino acid linker “TVAAP” (Zhang et al., 2024), named AF03-NL and AF03-IL respectively. AF03-IL containing a point mutation (Y27L) in IGF2, was named AF03-IL_m1_.

AF-03/AF03-IL/AF03-NL/AF03-IL_m1_ plasmid was transfected into CHO-S cells for expression and purification respectively and performed using the ÄKTA prime Plus system (GE Healthcare). MARV GP (Uganda strain, GenBank: AFV31370.1, aa 20–648, Δ277–455), CI-MPR1-3 (GenBank: NP_000867.3, aa36-466) and CI-MPR 11-13 (GenBank: NP_000867.3, aa 1508-1992) with six-histidine-tag at C-terminus were cloned into pcDNA3.1 vectors for expression in HEK293 cells respectively. The proteins were purified by nickel column (GE Healthcare, Cat. No. 11003399). SDS-PAGE and size exclusion chromatography was performed to identify the purity of recombinant proteins and mAbs.

### 2.2 SPR

The SPR (surface plasmon resonance) analysis was performed using a Biacore T200 machine with protein A chips (GE Healthcare) at room temperature. All the proteins used in SPR analysis were exchanged to BIAcore buffer (HBS-EP+ containing 0.03 M EDTA and 0.5% Surfactant P20, pH 7.4). The chip was subsequently immobilized with AF-03/AF03-NL/AF03-IL/AF03-IL_m1_ mAb (1 µg/ml) or IGF2 (1 µg/ml, Abcam, Cat. No. ab283420) respectively, ligand IGF2R 1-3/11-13 domain or IGF1R protein was diluted by running buffer at the concentrations ranging from 6.25 to 200 nM, and ligand MARV GP was diluted by running buffer at the concentrations ranging from 0.195 to 12.5 nM, associate 120 s and disassociate 280 s. The chip was regenerated with glycine-HCl (10 mM pH 2.0). Data were analyzed by Biacore T200 Evaluation Software.

### 2.3 Pseudovirus preparation

Pseudovirus were prepared as described previously (Zhang et al., 2024). In brief, HIV vector (pSG3.Δenv.cmvFluc) bearing EBOV, EBOV (parental and 3 mutants including A82V, T544I, A82V/T544I), SUDV, BDBV, TAFV, RESTV, BOMV, MARV, RAVN, MLAV, and LLOV GPs were prepared by liposome-mediated transfection of HEK293T cells using transfection reagent (JetPRIME), respectively. Cells were seeded in six-well plates at 7 × 10^5^ cells/well and transfected with 2 μg plasmids (0.4 μg GP and 1.6 μg HIV vector) when cells reached 60–80% confluence. Supernatants were collected at 48 h after transfection, centrifuged to remove cell debris at 3000 × g for 10 min, filtered through a 0.45 μm-pore filter (Millipore, SLHUR33RB), and stored at -80 °C.

### 2.4 Pseudovirus entry and antibody neutralization assay

For antibodies neutralization assays, HEK293T cells (3 × 10^4^ cells/100 μl/well) were seeded in 96-well plate and incubated overnight. Serial diluted AF-03/AF03-IL/ AF03-NL/ AF03-IL_m1_ antibodies (starting at 0.3 µM, threefold serially diluted, 50 μl/well) were incubated with cells at 37 °C for 2 h to enable internalization of the antibodies. In some settings, recombinant human IGF1R protein (Sino Biological, Cat. No. 10164-H08H-100) was pre-mixed with AF03-IL at 5:1 molar ratio for 30 min. Diluted pseudovirus (50 μl/well) was then added and incubated at 37 °C for 36 h. 100 μl of culture medium was discarded and the equal volume of Bright-Glo luciferase reagent (Vazyme, Cat. No. DD1204) was added in each well. Mixtures were transferred to 96-well whiteboards after 2 min reaction to detect the relative luciferase intensity in a fluorescence microplate reader (Promega).

### 2.5 Antibody attachment assay

HEK293T cells were harvested and incubated with 0.03, 0.06 and 0.12 µM of AF03-IL/AF03/NL/AF03-IL_m1_ antibody for 30 min at 4 °C, and unbound antibody was washed with PBS at 5000 × g, 1 min. PE anti-human IgG1 Fc antibody (Biolegend, Cat. No. 410708) was added and incubated for 30 min at 4 °C. Cells were collected for fluorescence detection on a FACSAria II flow cytometer (BD Biosciences). Data analysis was performed using the FlowJo software.

### 2.6 Evaluation of antibody internalization

HEK293T cells (500 μl/well, 1 × 10^5^ cells) were seeded into 48-well plates with cell density of 80% for experiments. Firstly, 5 μg/ml of antibody internalization reagent (Sartorius, Cat. No. 4722) was incubated with 100 μl of different concentrations of AF-03/AF03-IL/AF03-NL in dark for 20 min at 37 °C. The mixture was then added into HEK293T cells (1 × 10^5^/well), and incubated at 37 °C for 1, 3, 6, 12, 24, 48 h. Cells were harvested for flow cytometry. Data analysis was performed using the FlowJo software.

### 2.7 Antibody lysosomal localization assay

HEK293T cells (1 × 10^5^ per dish) were cultured overnight in a confocal dish pre-treated with polylysine (Beyotime, Cat. No. Y286384). A final concentration of 0.3 μM of internalization reagent (Sartorius, Cat. No. 4722) (100 μl) and 0.06 μM antibody (100 μl) were mixed, added to HEK293T cells and incubated for 6 h. Lysotracker Green (50 nM, Beyotime, Cat. No. C1047S) was added and incubated at 37 °C for 1 h. Hoechst 33342 (1 μg/ml, Beyotime, Cat. No. C1025) was incubated at 37 °C for 20 min and then imaged by Zeiss LSM880. Data analysis was performed using the ImarisViewer.

### 2.8 ELISA

HEK293T cells (1 × 10^5^/well) were seeded in 48-well plate. AF-03/AF03-IL/AF03-NL/AF03-IL_m1_ antibody (0.12 μM) was added and incubated at 37 °C respectively. At the indicated timepoint, supernatants were collected. MARV GP (2 µg/ml) was coated in coating buffer (0.1 M Na_2_CO_3_ and NaHCO_3_, pH 9.6) overnight at 4 °C. The plates were washed twice with PBST and then blocked with 4% nonfat milk at 37 °C for 1 h. The supernatants were added and incubated at 37 °C for 1 h. Subsequently, HRP-conjugated goat anti-human IgG (H + L) secondary antibody (1:6000, Thermo, Cat. No. 31130) was added and incubated at 37 °C for 30 min. Binding signals were visualized using a TMB substrate (CWBIO, Cat. No. CW0050), and the reaction was stopped by 2 N H_2_SO_4_. Absorbance at 450 nm was measured by a microplate reader.

### 2.9 Western blotting

Western blotting was performed as described previously (Zhang et al., 2022). Briefly, AF-03/AF03-IL/AF03-NL/AF03-IL_m1_ antibody (0.3 µM) was added and incubated with HEK293T cells at 37 °C respectively. At the indicated timepoint, cells were collected and then lysed in RIPA (Thermo, Cat. No. 89901) supplemented with protease inhibitor cocktail (Thermo, Cat. No.78429) for 30 min on ice. Lysis products were subjected to centrifugation. After centrifugation, each sample was loaded per lane using 4–12% SDS-PAGE and transferred to a PVDF membrane (Merck; IPVH00010). After blocking with 5% nonfat milk at room temperature for 1 h, the membrane was incubated with rabbit anti human heavy chain antibody (1:1000, Abcam, Cat. No. ab109489) at room temperature for 1 h. Immunoreactivity was detected using an enhanced chemiluminescence detection system (Chemiscope 6000, CLiNX).

### 2.10 IGF1R knockout

IGF1R and IGF2R were knockout as described previously with mild modification (Zhang et al., 2024). In brief, for lentivirus constructs, IGF1R/IGF2R sequence and lentiviral control plasmid were inserted into U6-sgRNA-EF1a-Cas9-FLAG-P2A-puro lentiviral vectors (genechem), respectively. The recombinant lentivirus was produced by co‐transfection of HEK293T cells with the plasmids U6-sgRNA-EF1a-Cas9-FLAG-P2A-puro, pHelper 1.0, and pHelper 2.0 with transfection reagents (genechem). Lentivirus‐containing supernatants were harvested 48 h after transduction and filtered through 0.22 μm cellulose acetate filters (Millipore, USA). Recombinant lentiviruses were then concentrated by ultracentrifugation (2 h, 50, 000 × *g*).

HEK293T cells (1 × 10^5^/well) were infected with IGF1R KO lentivirus and ctrl lentivirus (MOI=10) supplemented with polybrene at 37 °C for 16–24 h and then replaced with fresh medium, continued to be cultured for 48 h with addition of puromycin (2 μg/ml). 7–10 days later, cells were collected and IGF1R knockout was identified by flow cytometry.

### 2.11 Flow cytometry

Cells (1 × 10^6^) were stained on ice for 25 min with PE-conjugated anti-human IGF1R antibody (Biolegend, Cat. No. 351805) and FITC-conjugated anti-human IGF2R antibody (Biolegend, Cat. No. 364207) respectively, in PBS containing 2% heat-inactivated fecal calf serum. Subsequently, cells were collected for flow cytometry analysis as described above.

### 2.12 LC-MS/MS

The Coomassie-stained gel bands were excised and cut into 1 mm³ pieces, and then destained using 1 ml of 50% ACN / 50 mmol/l NH₄HCO₃ until complete discoloration. 1 ml of 100% ACN was added until the gel pieces shrank and turned white. ACN was discarded and the samples were air-dried at room temperature. Reduction was performed with 100 μl of 10 mmol/l DTT at 56 °C for 1 h, followed by alkylation using 100 μl of 20 mmol/l IAM in the dark at room temperature for 1 h. For in-gel digestion, 50 μl of trypsin (0.025 μg/μl) was added to fully absorb into the gel, followed by incubation at 37 °C for 16 h. Peptide extraction was carried out by adding 200 μl of extraction buffer (5% TFA, 50% CAN, 45% H₂O), incubating at 37 °C for 1 h, then sonicating for 5 min and centrifuging for 5 min. The supernatant was collected, and the extraction repeated once. Combined extracts were vacuum-dried at 57 °C. The liquid chromatography separation was performed on a reversed-phase analytical column (100 μm i.d. × 180 mm) packed with Reprosil-Pur 120 C18-AQ 3 μm particles. Mobile phase A consisted of 0.1% formic acid in water, and mobile phase B contained 0.1% formic acid in 80% acetonitrile. The flow rate was maintained at 400 nl/min throughout the 65-minute analytical method. The gradient elution program was set as following: 1% B at 0 min, increased to 8% B at 4 min, to 28% B at 53 min, and then to 100% B at 57 min, which was held until 60 min. The composition was subsequently returned to 1% B at 60.1 min and re-equilibrated until 65 min.

For mass spectrometric detection, full-scan MS spectra (Q Exactive HF-X Hybrid Quadrupole-Orbitrap Mass Spectrometer, Thermo Fisher Scientific) were acquired over the *m/z* range of 300–1800 with a resolution of 60,000. The automatic gain control (AGC) target was set to 3e6, and the maximum injection time (Max IT) was 20 ms. Precursor ions with intensity above 40 were selected for fragmentation via higher-energy collisional dissociation (HCD) with a normalized collision energy of 27%. MS/MS spectra were collected at a resolution of 15,000, with an AGC target of 5e4 and a Max IT of 22 ms. All raw data were recorded in raw format.

### 2.13 Statistical analysis

Data were analyzed, and the graphs were plotted using Prism software (GraphPad Prism 8). The data are presented as the mean ± standard error. Intergroup differences were compared using One or Two ANOVA. The *P* < 0.05 was considered statistically significant.

## 3 Results

### 3.1 Characterization of AF03-IL

**Fig. 1:**
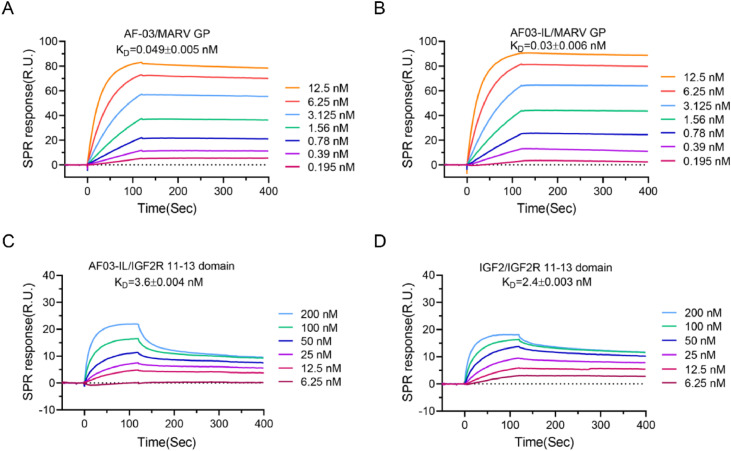
**AF03-IL shows fine binding potency to Marburg virus (MARV) glycoprotein (GP) as well as IGF2R segments.** (A,B) The binding kinetics of AF-03 (A) and AF03-IL (B) to MARV GP are detected by SPR assay. (C,D) The binding kinetics of AF03-IL (C) and IGF2 (D) to domain 11–13 of IGF2R protein are detected by SPR assay. The data were representative of three repeated experiments with similar results. Data are presented as mean ± SD.

To generate AF03-IL, We fused IGF2 protein to the N terminus of light chain of AF-03 via a peptide linker according to a protocol described previously (Wirchnianski et al., 2021). As shown in Fig. S1A&B, AF03-IL was expressed in eukaryotic cells. Its purity exceeded 95%. As controls, AF-03 and AF03-NL were also produced. Reducing SDS-PAGE of AF03-IL displayed three distinct bands with molecular weights ranged from 25 to 33 kDa. Mass spectrometry identified distinct light chain lengths corresponding to these bands (Table S1). We next measured the binding affinity of AF03-IL to MARV GP using SPR assay. The K_D_ value was 0.03±0.006 nM. This value was comparable to those of AF-03(0.049 ± 0.005 nM) and AF03-NL (0.047 ± 0.003 nM respectively) (Fig. 1A&B, S2A). Thus, IGF2 fusion did not significantly affect AF-03 binding to GP. We also measured binding affinity to the 11–13 domains of IGF2R protein. The K_D_ value was 3.6 ± 0.004 nM. This value resembled the affinity of free IGF2 for the same domains. Therefore, antibody coupling did not significantly affect IGF2 engagement with IGF2R (Fig. 1C&D). As expected, AF-03 and AF03-NL did not bind to these domains (Fig. S2B&C). In contrast, AF03-NL efficiently bound to 1–3 domains of IGF2R, whereas AF-03 and AF03-IL failed to interact with these regions (Fig. S2D-F).

### 3.2 The anti-filovirus activity of AF03-IL was much weaker than AF03-NL in vitro

**Fig. 2:**
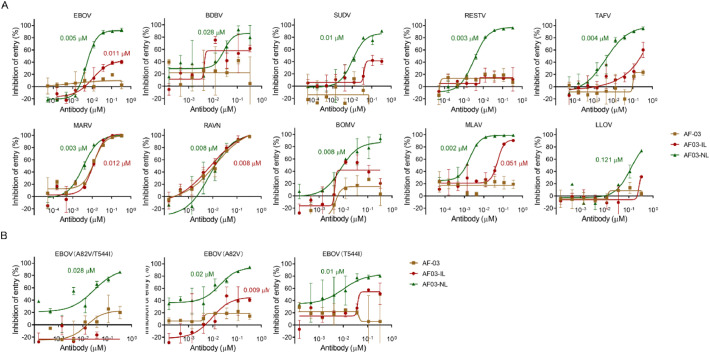
**The neutralizing activity of AF03-IL against pseudotypic filovirus infection is weaker that of AF03-NL.** (A) Pseudotyped filovirus as indicated is incubated with AF-03, AF03-IL, or AF03-NL at 37 °C for 1 h before infecting HEK293T cells. (B) Pseudotyped EBOV mutants are incubated with AF-03, AF03-IL, or AF03-NL respectively, and then infect HEK293T cells. Luciferase activity was measured and inhibition rates are calculated. The data were representative of three repeated experiments with similar results. IC_50_ values were shown.

We next tested the neutralizing capacity of AF03-IL in human cells. Recombinant HIV (Human immunodeficiency viruses) bearing GP from multiple filovirus species were used. AF03-IL markedly inhibited the entry of Marburgvirus species, including MARV and RAVN, into HEK293T cells. This inhibitory activity was comparable to that of AF-03 and AF03-NL (Fig. 2A). In sharp contrast, AF03-IL showed little or no neutralizing activity against other filovirus species. It also failed to neutralize three EBOV mutants. AF03-NL, however, exhibit broad-spectrum neutralization against all tested filoviruses (Fig. 2A&B). This result was unexpected. IGF2 is known as an alternative ligand for harnessing IGF2R-mediated endosomal delivery (Mikitiuk et al., 2023; Yang et al., 2024; Tian et al., 2024; Zhang et al., 2023).

### 3.3 AF03-IL was internalized poorly into cells compared with AF03-NL

**Fig. 3:**
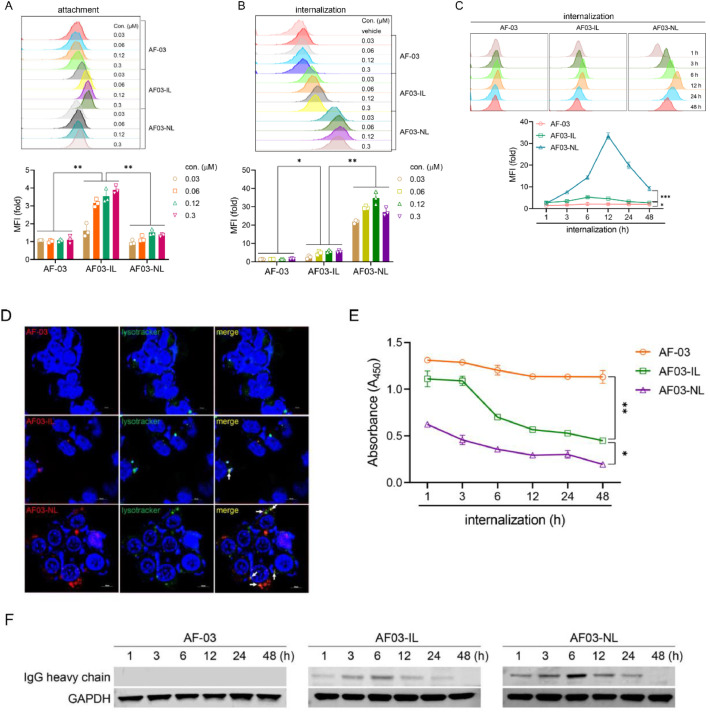
**The cellular internalization of AF03-IL is weaker compared with AF03-NL.** (A) AF-03, AF03-IL, or AF03-NL was incubated with cells at 4 °C for 1 hour. PE-conjugated secondary antibody was added and the fluorescence was detected by flow cytometry. (B,C) Dye-labeled AF-03, AF03-IL, or AF03-NL at the titrated concentrations (B) was incubated with cells at 37 °C for 1-48 h (C) to allow internalization. Fluorescence was detected by flow cytometry. Upper panels show representative plots or images; Lower panel show pooled data. (D) Dye-labeled AF-03, AF03-IL, or AF03-NL (0.06 µM) was incubated with cells at 37 °C for 3 h to allow internalization. The localization of antibodies in LE/LY was assayed by confocal microscopy. (E,F) AF-03, AF03-IL, or AF03-NL (0.06 µM) is incubated with cells at 37 °C for 1–48 h to allow internalization. Supernatants and cellular lysates were collected to analysis antibody levels by ELISA (E) and by Western blotting (F) respectively. Representative plots or images were shown. The data were pooled from at least three repeated experiments with similar results. Data are presented as mean±SD. Statistical significance was determined using the Two-way ANOVA. *p<0.05, **p<0.01, ***p<0.001.

It is well established that AF03-IL and AF03-NL bind to IGF2R. They are then endocytosed into the endosome or lysosome. This process inhibits filovirus entry into the cytoplasm (Zhang et al., 2024). We therefore investigated whether the weak neutralization potency of AF03-IL arose from impaired attachment or internalization. Unexpectedly, more AF03-IL attached to the cell surface than AF03-NL (Fig. 3A). In contrast, the amount of AF03-IL internalized into cells was much lower than that of AF03-NL. This was measured by flow cytometry and fluorescence microscopy respectively (Fig. 3B-D). Notably, internalization of both AF03-NL and AF03-IL peaked at 12 h after initiation. It then decreased dramatically (Fig. 3C). This suggests that the cargo was degraded in the lysosome. Consistent with this, cargo levels in the extracellular compartment gradually decreased once internalization began. This was true for both AF03-IL and AF03-NL. In contrast, AF-03 treatment did not reduce antibody reduction in the supernatants (Fig. 3E). Importantly, the decrease in cargo levels was more pronounced with AF03-NL than with AF03-IL (Fig. 3E). This indicates that less AF03-IL was transported into endosomal compartments. To directly detect internalized cargoes, we used an antibody against the heavy chain of human IgG for western blotting. Consistent with Fig. 3C, the antibody heavy chain was visible in cell lysates after AF03-IL or AF03-NL treatment. Intracellular levels first increased and then decreased (Fig. 3F). Taken together, these results show that AF03-IL has weak internalizing ability. This likely explains its poor neutralizing activity against filoviruses. To determine clearly the role of IGF2R in AF03-IL attachment and internalization, IGF2R in HEK293T cells was deleted (Fig. S3A). The results showed that IGF2R deletion augmented cellular attachment of the cargo, while weakening its uptake capability (Fig. S3B&C). Conversely, in IGF2R-overexpressing cells, the internalizing capacity of AF03-IL increases dramatically. It reached levels equal to AF03-NL (Fig. S4A&B). This suggests that the defect in AF03-IL internalization and neutralization is not caused by inherent structural features (e.g. AF-03-mediated steric occupancy to hinder IGF2:IGF2R interaction).

### 3.4 IGF2-IGF1R interaction hampers AF03-IL internalization and neutralization

**Fig. 4:**
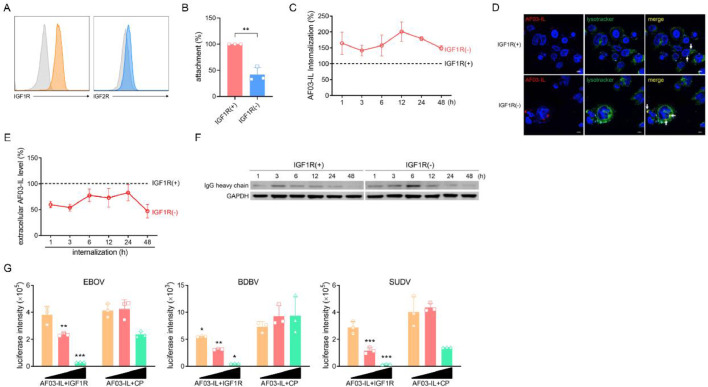
**The internalization and neutralization activity of AF03-IL are impaired by IGF2-IGF1R interaction.** (A) The surface expression of IGF1R and IGF2R on HEK293T cells was detected by flow cytometry. (B) AF03-IL (0.3 µM) was incubated with IGF1R-deficient or IGF1R-sufficient cells at 4 °C for 1 h. A PE-conjugated secondary antibody was added and fluorescence intensity was measured by flow cytometry. (C-F) AF03-IL (0.3 µM) was incubated with IGF1R-deficient or IGF1R-sufficient cells at 37 °C for 1-48 h to allow internalization. Fluorescence was detected by flow cytometry (C). Antibodies localization in LE/LY compartments was assayed by confocal microscopy (D). Supernatants were collected for quantification of antibody levels by ELISA (E). Cellular lysates were pooled for antibody detection by Western blotting (F). (G) AF03-IL was mixed with recombinant human IGF1R protein or control protein (CP), then incubated with pseudotyped ebolavirus species, followed by infecting HEK293T cells. Luciferase is assayed and inhibition rates are calculated. Representative plots or images were shown. The data were pooled from at least three repeated experiments with similar results. Data are presented as mean±SD. Statistical significance was determined using the Two-way ANOVA. *p<0.05, **p<0.01, ***p<0.001.

IGF2 can interact with three different receptors. It binds IGF1R and IGF2R with similar high affinity. It binds InsR with lower affinity (Roth et al., 1988; Zhu et al., 2022; Belfiore et al., 2023). We therefore hypothesized that AF03-IL activity was affected by IGF2 interaction with IGF1R. Flow cytometry analysis verified that IGF1R was abundantly expressed on the cell surface at significantly higher levels than IGF2R (Fig. 4A). AF03-IL bound to IGF1R with nanomolar affinity (Fig. S5). To directly test the role of IGF1R, we generated IGF1R-knockout cells, IGF2R expression was not affected. This was confirmed by flow cytometry (Fig. S6). Intriguingly, AF03-IL attachment was dramatically abolished in IGF1R-deficient cells (Fig. 4B). This implies that AF03-IL attachment is mainly mediated by IGF2 binding to IGF1R, not to IGF2R. Conversely, IGF1R deletion markedly increased AF03-IL internalization (Fig. 4C&D). Consistent with this, extracellular AF03-IL levels decreased. Intracellular heavy chain contents increased significantly. These changes were observed compared to IGF1R-sufficient cells (Fig. 4E&F). However, entry of ebolavirus species into IGF1R-deficient cells was impaired. This was observed compared to IGF1R-sufficient counterparts (Fig. S7). This finding agrees with a previous study (Stewart et al., 2021). Consequently, AF03-IL neutralizing activity against ebolavirus species was not enhanced in IGF1R-deficient cells (data not shown). Alternatively, we then blocked IGF2-IGF1R interaction by adding recombinant IGF1R protein. Pre-mixing AF03-IL with IGF1R protein significantly enhanced its neutralizing ability against ebolavirus species (Fig. 4G). Taken together, these results demonstrate that IGF2-IGF1R interaction strongly impacts AF03-IL internalization and neutralization.

### 3.5 AF03-IL_m1_ bearing one point mutation exhibited superior internalization and neutralization capacity

**Fig. 5:**
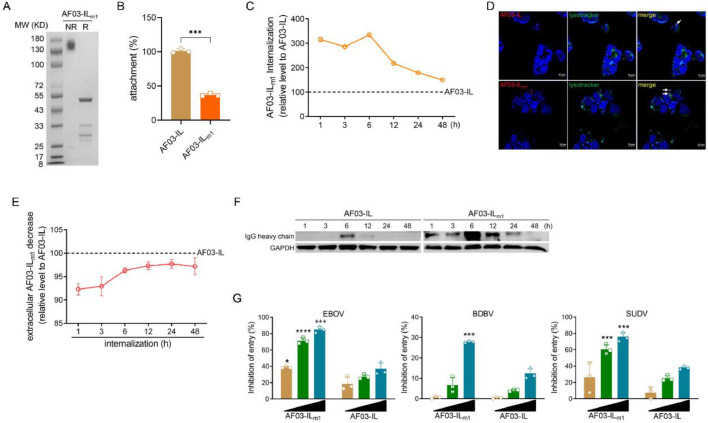
**IGF2 point mutation confers superior internalization and neutralization potency of AF03-IL.** (A) AF03-IL_m1_ is prepared and characterized by SDS-page. (B) AF03-IL or AF03-IL_m1_ (0.3 µM) is incubated with cells at 4 °C for 1 h. PE-conjugated secondary antibody is added and the fluorescence is detected by flow cytometry. (C-F) The AF03-IL or AF03-IL_m1_ (0.3 µM) is incubated with HEK293T cells at 37 °C for 1-48 h to allow internalization. The fluorescence is detected by flow cytometry (C). The localization of antibodies in LE/LY is assayed by confocal microscopy (D). The supernatants are collected for analysis of antibody contents by ELISA (E). The cellular lysates are pooled for antibody detection by western blotting (F). (G) Pseudotyped ebolavirus species are incubated with AF03-IL or AF03-IL_m1_ respectively, and then infects HEK293T cells. Luciferase is assayed and inhibition rates are calculated. Representative plots or images were shown. The data were pooled from at least three repeated experiments with similar results. Data are presented as mean±SD. Statistical significance was determined using the Two-way ANOVA. *p<0.05, ***p<0.001, ****p<0.0001.

To overcome the obstacle described above, we introduced a single point mutation into the IGF2 segment of AF03-IL (Fig. 5A). Specifically, we substituted Leu^27^ with Tyr^27^. This substitution rendered the protein unable to bind IGF1R (Fig. S8A). Meanwhile, it maintained high affinity for MARV GP and for the 11-13 domain of IGF2R (Fig. S8B&C). These findings are consistent with previous reports (Beukers et al., 1991; Roth et al., 1991). AF03-IL_m1_ showed poor attachment compared to parental AF03-IL (Fig. 5B). This result further supports the notion that attachment depends on IGF2 binding to IGF1R. Importantly, substantially more AF03-IL_m1_ was internalized (Fig. 5C&D). This was accompanied by a remarkable decreased of cargo in the supernatants. It was also accompanied by increased intracellular levels of IgG heavy chain (Fig. 5E&F). Compared to the parental construct without the mutations, AF03-IL_m1_ exhibited stronger neutralizing ability against ebolavirus species (Fig. 5G). It should be noted that the enhanced broad-spectrum neutralization of AF03-IL_m1_ is not attributed to altered viral GP-binding specificity. Instead, the Y27L mutation ameliorates IGF1R-mediated cell surface retention, improves receptor-dependent internalization, and thereby unmasks the inherent cross-reactive neutralizing activity of the parental AF-03 antibody. Nevertheless, despite the obvious functional improvement of AF03-IL_m1_ relative to AF03-IL, its overall internalization and neutralization potency are still inferior to those of the NPC2-fused AF-03.

## 4 Discussion

In this study, we demonstrated that the IGF2-fused antibody AF03-IL had much weaker pan-filovirus neutralize capacity than AF03-NL. This suggests that IGF2 attachment may not be an optimal alternative for developing broad-spectrum anti-filovirus therapeutics based on scavenger-directed endosomal delivery. This finding is unlikely due to the specific antibody used in this work. A previous study reported similar results when IGF2 was fused to another anti-filovirus antibody (Wirchnianski et al., 2021). Importantly, introducing Y27L mutation markedly improved internalization and neutralization of this cargo. The Y27L mutation abrogates binding to IGF1R while preserving high-affinity recognition of IGF2R. This mechanistic interpretation is fully supported by previously reported structural and functional evidence. Sakano et al. first validated that mutagenesis at IGF2 residue 27 effectively remodeled receptor selectivity, switching the intrinsic receptor-binding preference of IGF2 (Sakano et al., 1991). Subsequent structural and functional studies further confirmed that residue 27 resides at the critical interfacial region was exclusively responsible for IGF1R interaction. Mutation at this position eliminated the key molecular contacts required for stable IGF1R binding, whereas the independent IGF2R-binding epitope remained structurally intact and functionally unperturbed (Brown et al., 2008; Forbe et al., 2001). Endocytosis mediated by the IGF2-IGF2R axis represents a canonical pathway for the lysosomal degradation of extracellular and membrane proteins (Mikitiuk et al., 2023; Yang et al., 2024; Tian et al., 2024; Zhang et al., 2023; Pan et al., 2025). Consistent with this concept, the improved neutralization potency of AF03-IL_m1_ is directly attributable to elevated cellular internalization. Notably, the Y27L mutation does not alter the binding affinity for viral glycoproteins (Fig. S8B), excluding any mutation-induced alteration in viral target recognition. Our findings may also provide valuable mechanistic implications for the rational design of lysosome-targeting chimeras (LYTACs) and other IGF2-based lysosomal delivery tools. Further biochemical and cellular studies are still required to fully validate this translational possibility and optimize related targeting strategies.

It is noted that the Y27L mutation eliminates IGF1R interference with IGF2-IGF2R binding. This mutation significantly improves internalization and neutralization of AF03-IL. However, the modified cargo remains weaker than the NPC2-fused AF-03. Several reasons may explain this observation: First, IGF2 is present in fetal bovine serum during cell culture (Fig. S9). This may compete with AF03-IL_m1_ for binding to IGF2R. Consequently, it may partially compromise the internalization capacity of AF03-IL_m1_. This issue could be resolved, at least in part, by introducing gain-of-function mutations that enhance affinity for IGF2R. Such an approach was recently reported (Yang et al., 2024). Accordingly, we generated a new cargo carrying four mutations (E6R/F19L/Y27L/S39P). This construct was named AF03-IL_m4_ (Fig. S10A). Unexpectedly, AF03-IL_m4_ showed lower affinity for IGF2R (Fig. S10B). One possible explanation is that Y27L mutation may impair binding of the three-mutated IGF2 to IGF2R. Alternatively, antibody fusion may greatly affect interaction between the four-mutated IGF2 and IGF2R. Second, IGF2 activity is controlled by IGF-binding proteins (IGFBPs). IGFBP3 and IGFBP6 are particularly important. They determine protein availability (Chao and D'Amore, 2008). We detected IGFBP6 in the cell supernatants (Fig. S11). This suggests that cargo function may be compromised by IGFBP6 binding, which could hinder the interaction between the cargo and IGF2R. Finally, the insulin receptor (InsR) expression was detected on the surface of HEK293T cells (Fig. S12). InsR is another receptor for IGF2 (An et al., 2024; Andersen et al., 2017), its expression may interfere with the function of AF03-IL, which deserves further investigation in future work. We believe that structure-guided or computational design approaches could be leveraged to refine receptor binding specificity. Moreover, in silico modeling of IGF2–receptor interactions may help identify mutations that preserve uptake via the intended pathway while minimizing off-target binding.

Several pipelines have been reported for developing broad-spectrum anti-filovirus antibodies. These include bispecific or trispecific antibodies that cross-react with two different epitopes on GP (Nyakatura et al., 2018; Frei et al., 2016; Wirchnianski et al., 2024). They also include antibody cocktails (Liu et al., 2023) and Trojan horse antibodies (Wec et al., 2016; Wirchnianski et al., 2021). In this study, we used the Trojan horse strategy to confer broad-spectrum activity of AF-03 against filovirus species with distinct IC_50_ values. A plausible explanation is that the AF-03 epitopes are not highly conserved across the filovirus family. Therefore, future work should focus on optimizing epitope selection. Screening for novel antibody against conserved sequence within the RBD is also warranted. Due to limited access to BSL-4 facilities, authentic live filovirus validation was not feasible here and remains for future study. Besides, all assays in this study were performed in one cell line with fixed IGF1R/IGF2R levels. Testing more cell types with different receptor ratios will help generalize our conclusions.

## Data availability statements

The data underlying this article are available in the article and in its online supplementary material.

## Patient consent statement

The study does not include factors necessitating patient consent.

## Funding statement

This work was supported by The 10.13039/501100001809National Natural Science Foundation of China grant number 31771010 and 81672803.

Postgraduate Research & Practice Innovation Program of Jiangsu Province (Grant No. KYCX25_4333), 10.13039/501100012216Jiangsu University of Science and Technology, China.

## CRediT authorship contribution statement

**Wen Hu:** Writing – original draft, Visualization, Investigation, Formal analysis. **Xiaonan Wu:** Writing – original draft, Validation, Investigation, Formal analysis. **Yuting Zhang:** Investigation, Formal analysis. **Siyu Zhao:** Investigation, Formal analysis. **Junjie He:** Investigation. **Xuanyi Li:** Investigation. **Muyang He:** Investigation. **Jing Wang:** Investigation. **Longlong Luo:** Investigation. **He Xiao:** Investigation. **Xinying Li:** Investigation. **Chenghua Liu:** Investigation. **Weijin Huang:** Formal analysis, Conceptualization. **Youchun Wang:** Formal analysis, Conceptualization. **Chunxia Qiao:** Formal analysis, Conceptualization. **Jiannan Feng:** Supervision, Formal analysis, Conceptualization. **Yan Wen:** Investigation. **Guojiang Chen:** Writing – review & editing, Writing – original draft, Supervision, Investigation, Funding acquisition, Formal analysis, Conceptualization.

## Declaration of competing interest

The authors declare that no conflict of interests exists.

## Appendix Supplementary materials

Image, application 1

Image, application 2

Image, application 3
