# Supplementary material for: Pan-filovirus activity of an IGF2-fused monoclonal antibody: Impairment by IGF1R cross-engagement and rescue via IGF2 Y27L mutation
Source: Virus Res. 2026 May 25;369:199755. doi: 10.1016/j.virusres.2026.199755 (PMC13234713; doi:10.1016/j.virusres.2026.199755)

**Supplementary Figure legends**

**Figure S1. The characteristics of AF03-IL.** (A) AF-03 and AF03-IL are prepared and characterized by SDS-page. (B) The purity of AF-03, AF03-IL, AF03-NL is determined by SEC assay. The data were representative of two repeated experiments with similar results.

**Figure S2. SPR assays.** The binding kinetics of AF03-NL to MARV GP (A), AF-03 to IGF2R 11-13 domain (B), AF03-NL to IGF2R 11-13 domain (C), AF03-NL to IGF2R 1-3 domain (D), AF-03 to IGF2R 1-3 domain (E), AF03-IL to IGF2R 1-3 domain (F) is detected by SPR. The data were representative of two repeated experiments with similar results.

**Figure S3. The weakened internalizing ability of AF03-IL in IGF2R-KO cells.** (A) HEK293T cells were infected with CAS9-KO lentivirus targeting human IGF2R. On day 7 post infection, IGF2R expression on the cell surface is detected by flow cytometry. (B) AF03-IL was incubated with cells at 4℃ for 1 hr. PE-conjugated secondary antibody was added and the fluorescence was detected by flow cytometry. (C) The dye-labeled AF03-IL or AF03-NL at 0.06 µM is incubated with HEK293T cells at 37℃ for 6 hr to allow internalization. The fluorescence is detected by flow cytometry. The plots were representative of three repeated experiments with similar results.

**Figure S4. The enhanced internalizing ability of AF03-IL in IGF2R-overexpressing cells.** (A) IGF2R plasmid is introduced transiently into HEK293T cells. IGF2R expression was detected by flow cytometry at 48 hr. (B) The dye-labeled AF03-IL or AF03-NL at 0.06 µM is incubated with HEK293T cells at 37℃ for 6 hr to allow internalization. The fluorescence is detected by flow cytometry. The plots were representative of three repeated experiments with similar results.

**Figure S5. SPR assays.** The binding kinetics of AF03-IL to IGF1R is detected by SPR. The data were representative of two repeated experiments with similar results.

**Figure S6. The expression of IGF1R and IGF2R in IGF1R-knockout cells is determined.** HEK293T cells were infected with CAS9-KO lentivirus targeting human IGF1R. On day 7 post infection, IGF1R and IGF2R expression on the cell surface is detected by flow cytometry respectively. The plots were representative of two repeated experiments with similar results.

**Figure S7. IGF1R deletion weakened the cellular entry of ebolavirus species.** Pseudotypic ebolavirus species infects IGF1R–deficient or sufficient cells respectively. Luciferase is assayed. The data were pooled from three repeated experiments with similar results. Data are presented as mean±SD. Statistical significance was determined using the Two-way ANOVA. **p<0.05, **p<0.01*.

**Figure S8. SPR assays.** The binding kinetics of AF03-IL_m1_ to IGF1R (A), AF03-IL_m1_ to MARV GP (B), AF03-IL_m1_ to IGF2R 11-13 domain (C) is detected by SPR. The data were representative of three repeated experiments with similar results.

**Figure S9. Extracellular and intracellular IGF2 contents.** The supernatants and cell lysates were collected respectively. IGF2 is detected by western blotting. The data were representative of two repeated experiments with similar results.

**Figure S10. The binding affinity of AF03-IL_m4_ to IGF2R is determined.** (A) AF03-IL_m4_ is prepared and characterized by SDS-page. (B) The binding kinetics of AF03-IL_m4_ to IGF2R 11-13 domain is detected by SPR. The data were representative of three repeated experiments with similar results.

**Figure S11. Extracellular and intracellular IGFBP3/6 contents.** The supernatants and cell lysates were collected respectively. IGF2BP3/6 is detected by western blotting. The images were representative of two repeated experiments with similar results.

**Figure S12. The expression of InsR in HEK293T cells is determined.** InsR expression on the cell surface of HEK293T cells is detected by flow cytometry. The plots were representative of two repeated experiments with similar results.

**Table S1. Determination of protein identity by LC MS plus MS.** Mass spectrometry analysis identified the distinct length of the light chain of AF03-IL in these bands respectively. The data were representative of two repeated experiments with similar results.

Fig. S1


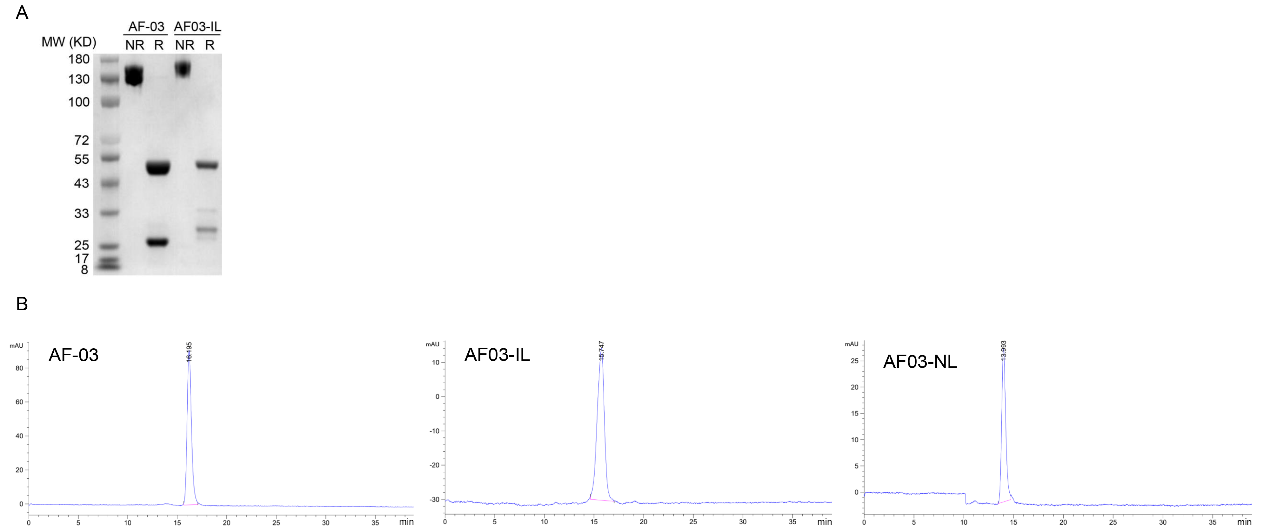


Fig. S2


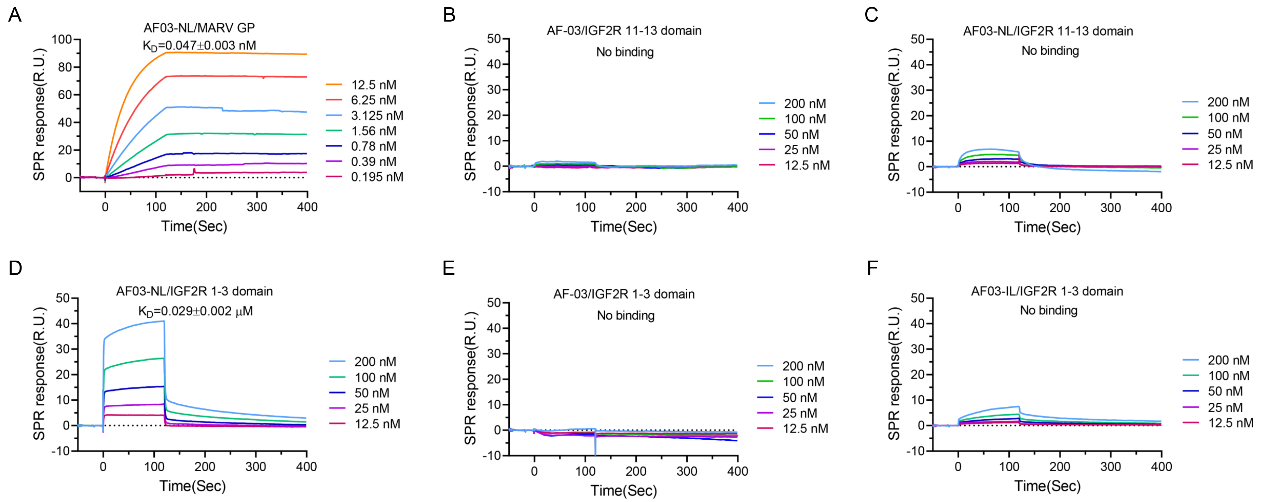


Fig. S3


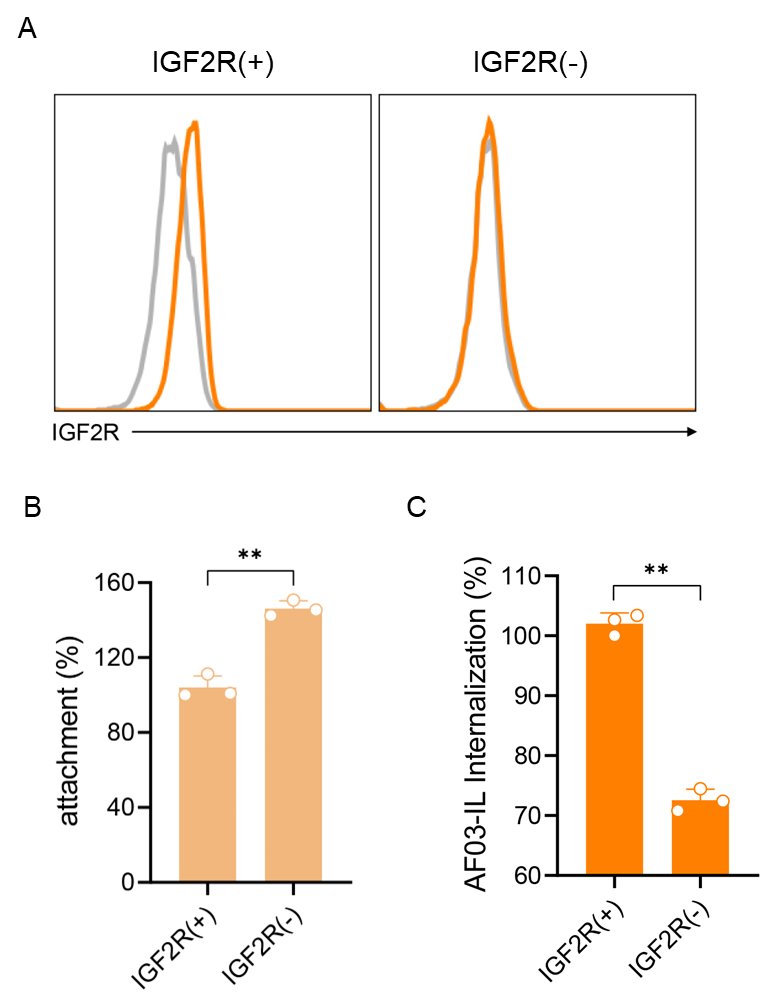


Fig. S4


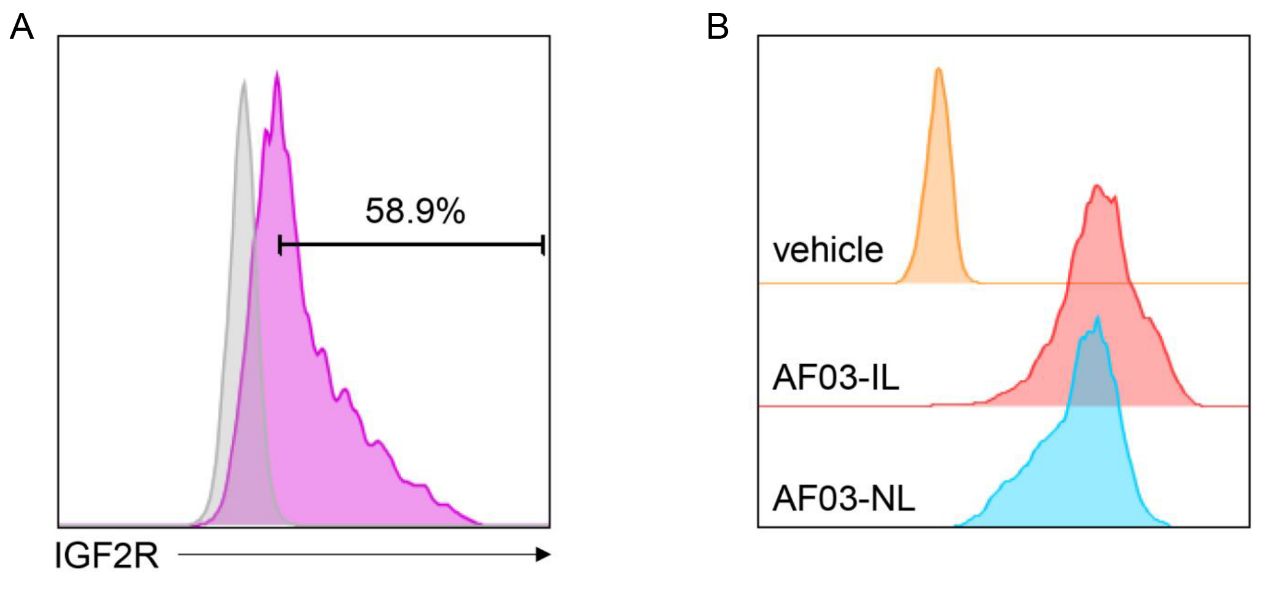


Fig. S5


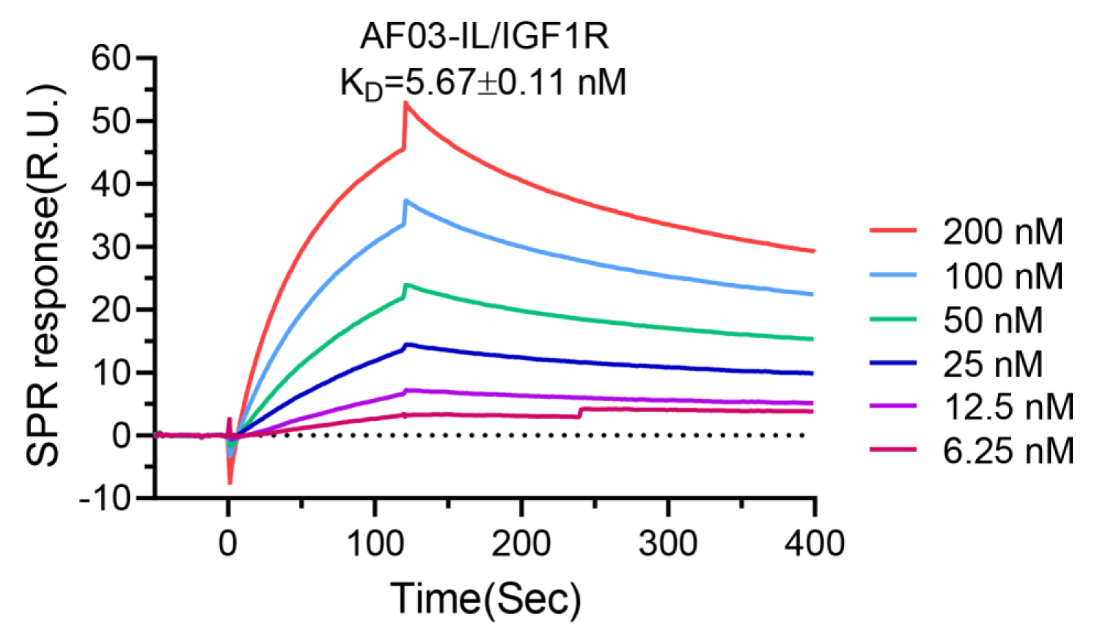


Fig. S6


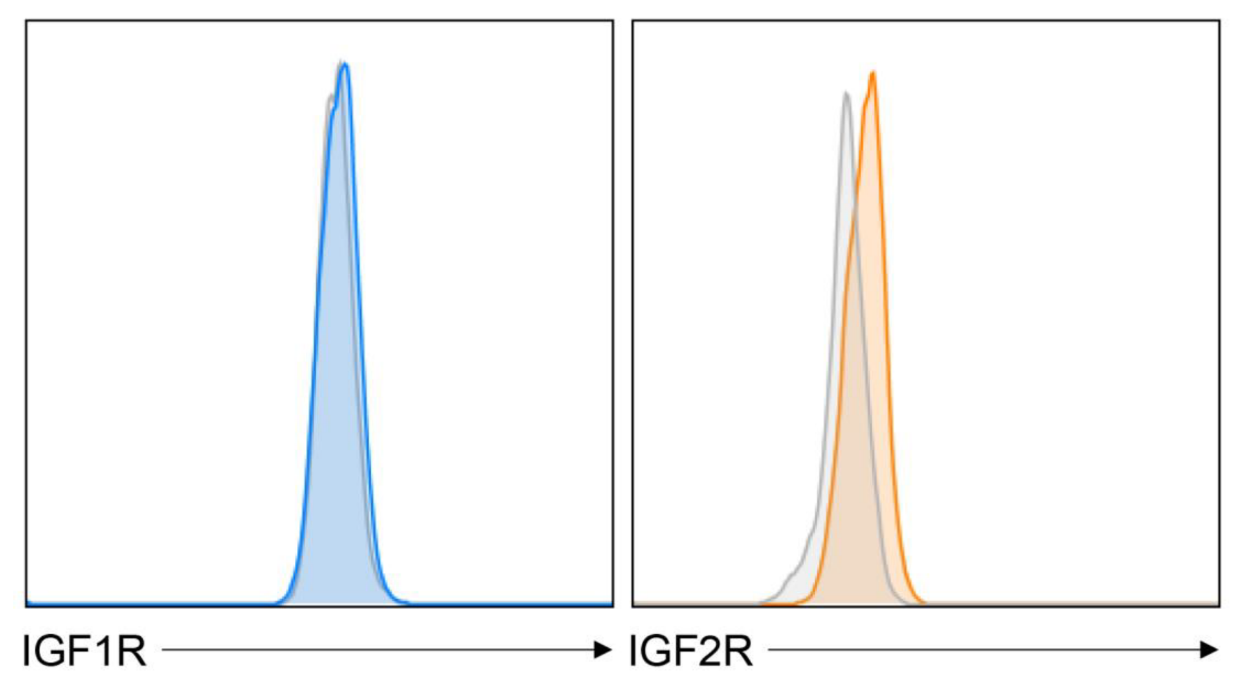


Fig. S7


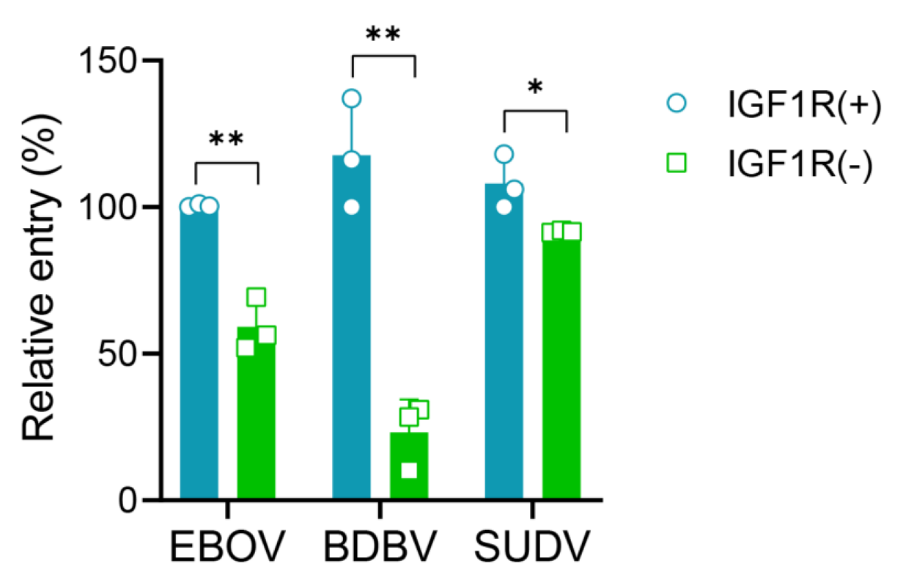


Fig. S8


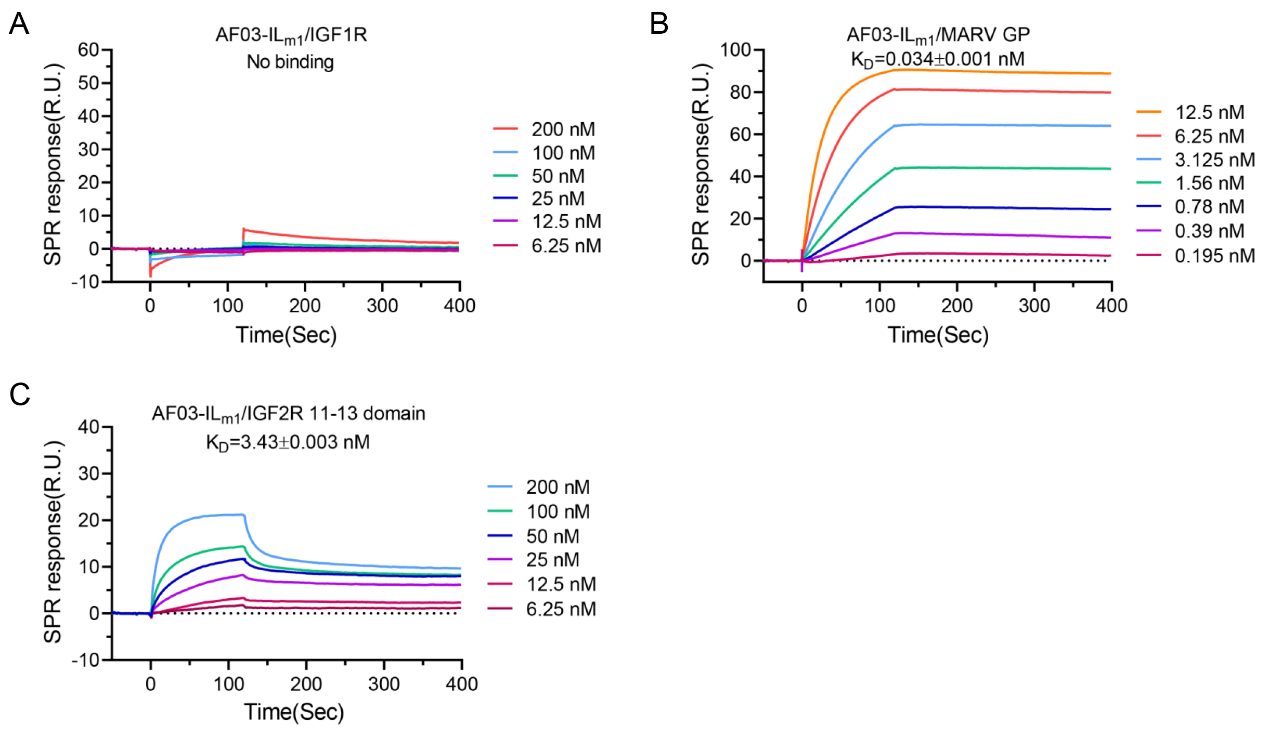


Fig. S9


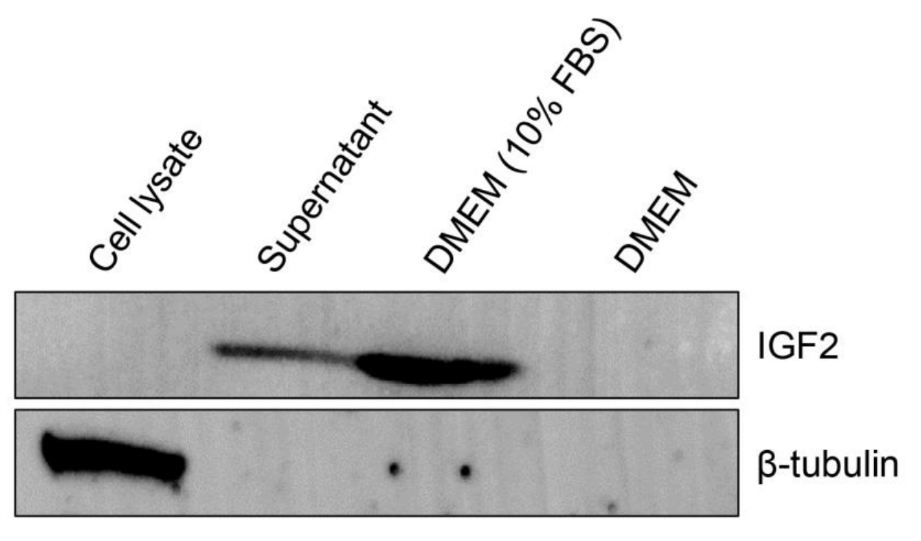


Fig. S10


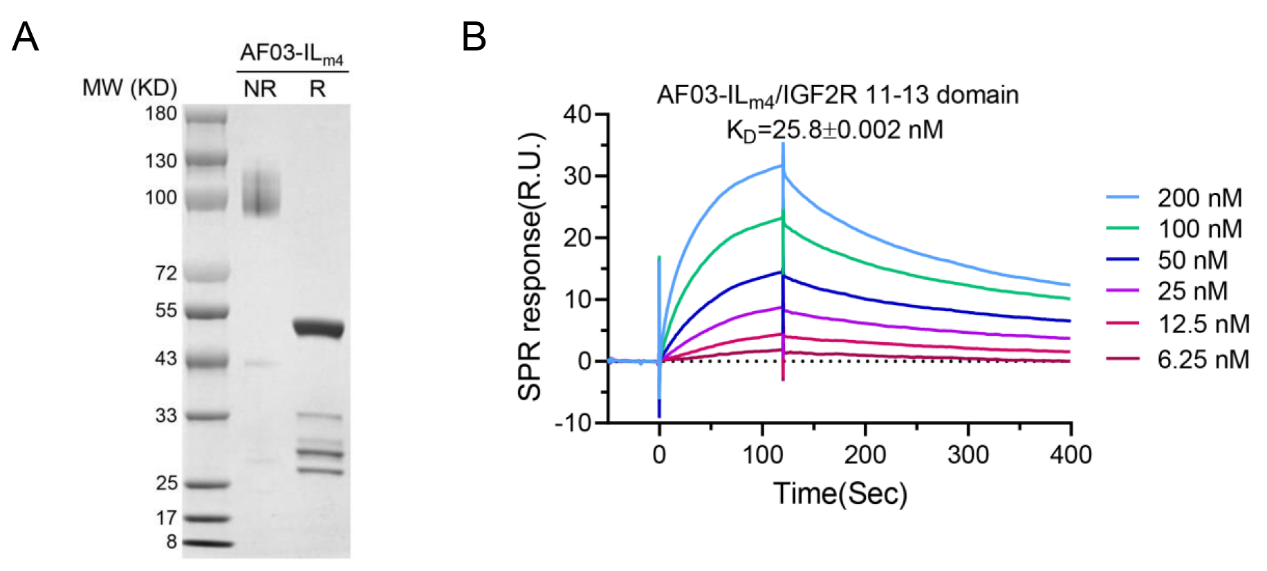


Fig. S11


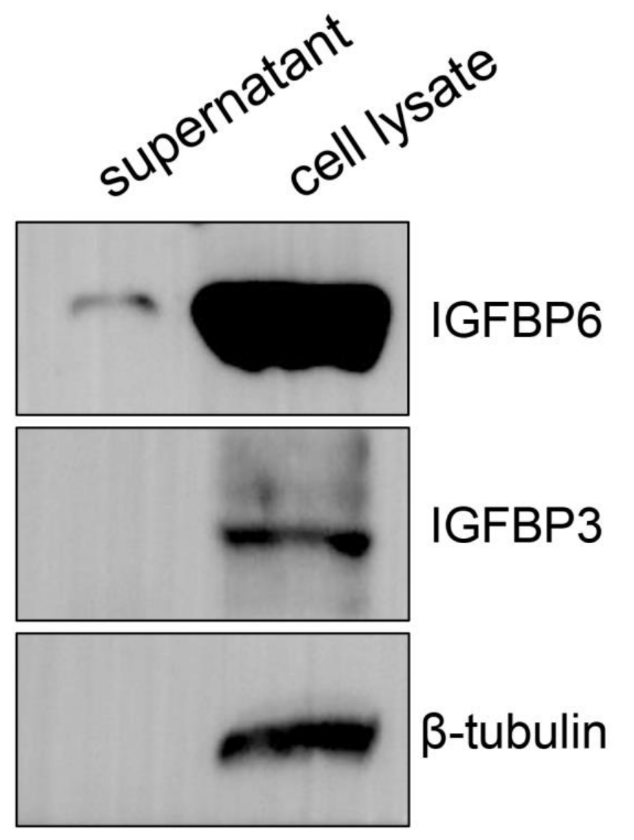


Fig. S12


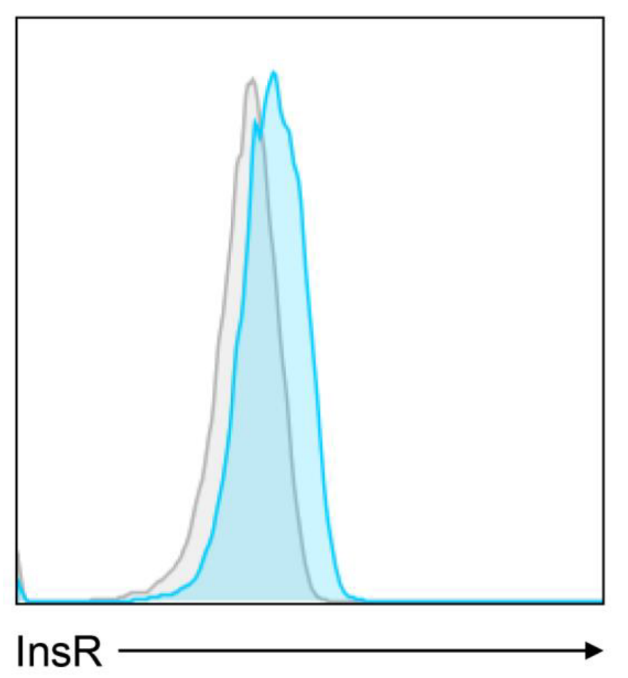

Supplement: Supplementary file 1 [file mmc1.docx]
